# Acute kidney injury in patients with pulmonary embolism: A population-based cohort study

**DOI:** 10.1097/MD.0000000000005822

**Published:** 2017-03-03

**Authors:** Chih-Hsiang Chang, Chung-Ming Fu, Pei-Chun Fan, Shao-Wei Chen, Su-Wei Chang, Chun-Tai Mao, Ya-Chung Tian, Yung-Chang Chen, Pao-Hsien Chu, Tien-Hsing Chen

**Affiliations:** a Department of Nephrology, Kidney Research Center, Chang Gung Memorial Hospital, Linkou Medical Center; b Department of Nephrology, Chang Gung Memorial Hospital, Kaohsiung Medical Center; c Division of Thoracic and Cardiovascular Surgery, Department of Surgery, Chang Gung Memorial Hospital, Linkou Medical Center, Chang Gung University, Taoyuan City; d Department of Cardiology, Chang Gung Memorial Hospital, Linkou Medical Center; e Clinical Informatics and Medical Statistics Research Center, Chang Gung University, Taoyuan City; f Department of Cardiology, Chang Gung Memorial Hospital, Keelung Branch; g Division of Allergy, Asthma, and Rheumatology, Department of Pediatrics, Chang Gung Memorial Hospital, Taoyuan, Taiwan.

**Keywords:** acute kidney injury, database, prognosis, pulmonary embolism, risk factors

## Abstract

Acute kidney injury (AKI) is overlooked in patients with pulmonary embolism (PE). Risk factors for and long-term outcomes of this complication remain unknown. This study evaluated the predictors and prognosis of AKI in patients with PE.

This retrospective cohort study used Taiwan's National Health Insurance Research Database. We enrolled a total of 7588 patients who were admitted to a hospital for PE from January1997 to December 2011 and administered anticoagulation or thrombolytic agents. All demographic data, risk factors, and outcomes were analyzed.

AKI was diagnosed in 372 (4.9%) patients. Multivariate logistic regression analysis revealed pre-existing chronic kidney disease, hypertension, diabetes mellitus, massive PE, anemia, and sepsis as independent risk factors for AKI. In the long-term follow-up, the survival rate was similar in the AKI and non-AKI groups.

Careful risk factor screening and intensive intervention in patients with AKI might yield outcomes similar to those in patients without AKI.

## 1 Introduction

Acute kidney injury (AKI) is associated with mortality and complications in hospital settings, with an incidence of 28% to 75% based on different etiologies.^[1–5]^ AKI is also reported to be associated with long-term mortality and dialysis dependence. In patients with pulmonary embolism (PE), renal dysfunction was not rare, with an incidence of 5.1% to 13%.^[6,7]^ h.4d34og8 Goldhaber et al^[7]^ used the ICOPER registry including 2454 patients and reported that the incidence of renal dysfunction, defined as Cr > 2.0 mg/dL, was 5.1% in patients with PE, and renal dysfunction itself was an independent predictor of mortality (HR, 2.0; 95% CI, 1.4–3.0). Kostrubiec et al^[6]^ conducted a study involving 100 patients with PE and reported renal failure, defined as Cr > 1.5 mg/dL, in 13 patients (13%). The HR of renal failure-related mortality was 6.4 (95% CI, 2.22–18.61). In another study, Kostrubiec et al enrolled 142 patients with PE and evaluated the eGFR, plasma neutrophil gelatinase-associated lipocalin (NGAL), and cystatin-C at admission to detect kidney injury.^[8]^ Nonsurvivors had lower eGFR and higher NGAL, which predicted the 30-day mortality. AKI was reported in 31% of patients on the basis of NGAL exceeding 70 ng/mL. In that study, the eGFR was weakly correlated with the left ventricle ejection fraction and tricuspid regurgitation. No previous study has focused on the risk factors for and long-term outcomes of AKI. Thus, this study identified the predictors of AKI in the aforementioned population. The present findings may guide physicians in the management of patients with AKI after PE.

## 2 Materials and Methods

### 2.1 Study design, patient information, and data collection

**Figure 1:**
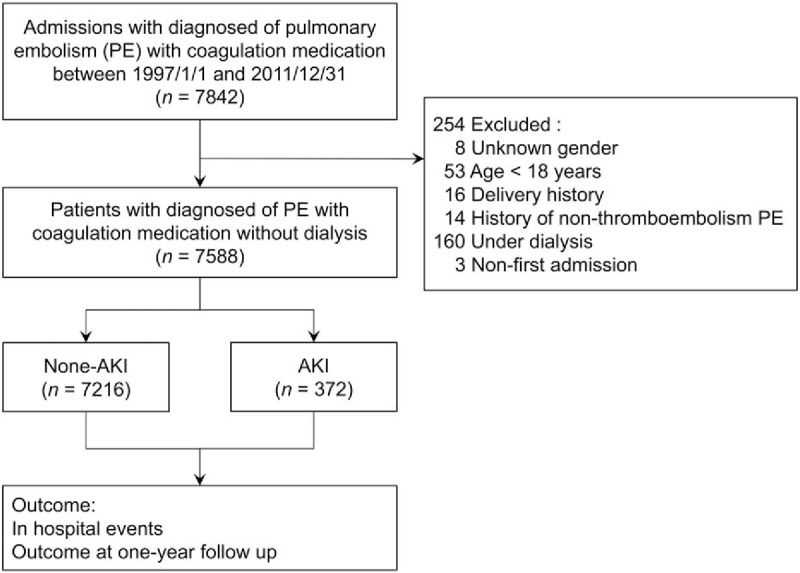
Patient enrollment flowchart. Patients hospitalized with a diagnosis of PE and administered anticoagulants were included in our analysis after relevant exclusions. PE = pulmonary embolism.

Data for this open-label, longitudinal, analytical cohort study were retrieved from Taiwan's National Health Insurance Research Database (NHIRD), one of the largest prospective collective datasets.^[9]^ This study was conducted on the basis of the Declaration of Helsinki and was approved by the ethics committee of the Institutional Review Board of Chang Gung Memorial Hospital. The study period was from January 1, 1997, to December 31, 2011. We enrolled patients who received a diagnosis of PE in the NHI admission dataset (DD).The patient eligibility criteria were as follows: (1) aged more than 18 years, (2) no history of PE, (3) nonperipartum PE, and (4) heparin or low-molecular-heparin administration during hospitalization. We defined PE admission as the index admission. We classified the study population into the AKI and non-AKI groups (Fig. 1). AKI diagnosis was coded according to the International Classification of Diseases, Ninth Revision (ICD-9) 584 and dialysis on the index admission. We recorded events on the index admission and at the 1-year follow-up. The follow-up was continued until December 31, 2011, or death.

### 2.2 Definitions: comorbidities and outcomes

We confirmed comorbidities on the basis of the original claims DD. We retrieved malignancy diagnosis from the Registry of Catastrophic Illness Patient Database, an NHIRD subset, and positive tissue proof of malignancy from the NHIRD. Massive PE was detected according to the following criteria: (1) positive PE and (2) hemodynamic disturbance requiring inotropic agents, including dopamine, norepinephrine, and epinephrine.^[10]^ Moreover, traumatic injury was detected according to the following:(1) traumatic diagnosis and (2) positive operations from the original reimbursement data. Anemia was defined as the diagnosis and need for transfusion of more than 2U of blood at admission. Sepsis was defined as proven or suspected infections and the presence of 2 or more systemic inflammatory response syndrome criteria. CKD was defined as an estimated glomerular filtration rate (eGFR) of less than 60 mL/min. Short-term outcomes were defined as in-hospital events involving the kidney and critical, cardiovascular, and bleeding events. De nova AKI requiring hemodialysis is patient had AKI and hemodialysis in index admission. Heart failure (HF), myocardial infarction (MI), and pulmonary hypertension diagnoses were based on ICD-9 codes in the NHIRD DD. Major bleeding events included intracerebral hemorrhage and gastrointestinal bleeding. Furthermore, the 1-year outcome in patients included acute events requiring hospitalization, including deep vein thrombosis (DVT), PE, HF, and major bleeding. We designed the following inclusion criteria for patients who were diagnosed as having dialysis dependence (1) low eGFR, typically less than 5 cc/min;(2)structural changes in CKD, such as contracted kidneys, as evaluated through echorenography; and (3)an irreversible deterioration of renal function for more than 6 months.

### 2.3 Statistical analysis

The clinical characteristics of patients in the AKI and non-AKI groups were compared using the chi-squared test for categorical variables and Student's *t* test for continuous variables in univariate analysis. To determine the factors associated with the post-PE risk of AKI, we conducted a multivariate logistic regression analysis, in which variables significant (*P* < 0.05) in the univariate analyses were introduced into the multivariable model. The risk of in-hospital events between the groups was compared using multivariable logistic and Poisson regression analysis for binary (i.e., in-hospital mortality) and count (i.e., length of ICU stay) outcomes, respectively, with adjustments for 20 patient characteristics (Table 1). After excluding patients who died during the index admission, we compared the risk of time-to-event outcomes (i.e., recurrent PE) between the study groups by using a multivariable Cox proportional hazard model after adjustments for the aforementioned variables. Finally, we plotted the cumulative Kaplan–Meier survival curves of the selected time-to-event outcomes, including dialysis dependence, CKD, and all-cause mortality, and performed the log-rank test to compare group differences. We conducted the data analyses by using SPSS 22 (IBM Corp, Armonk, NY).

**Table 1:**
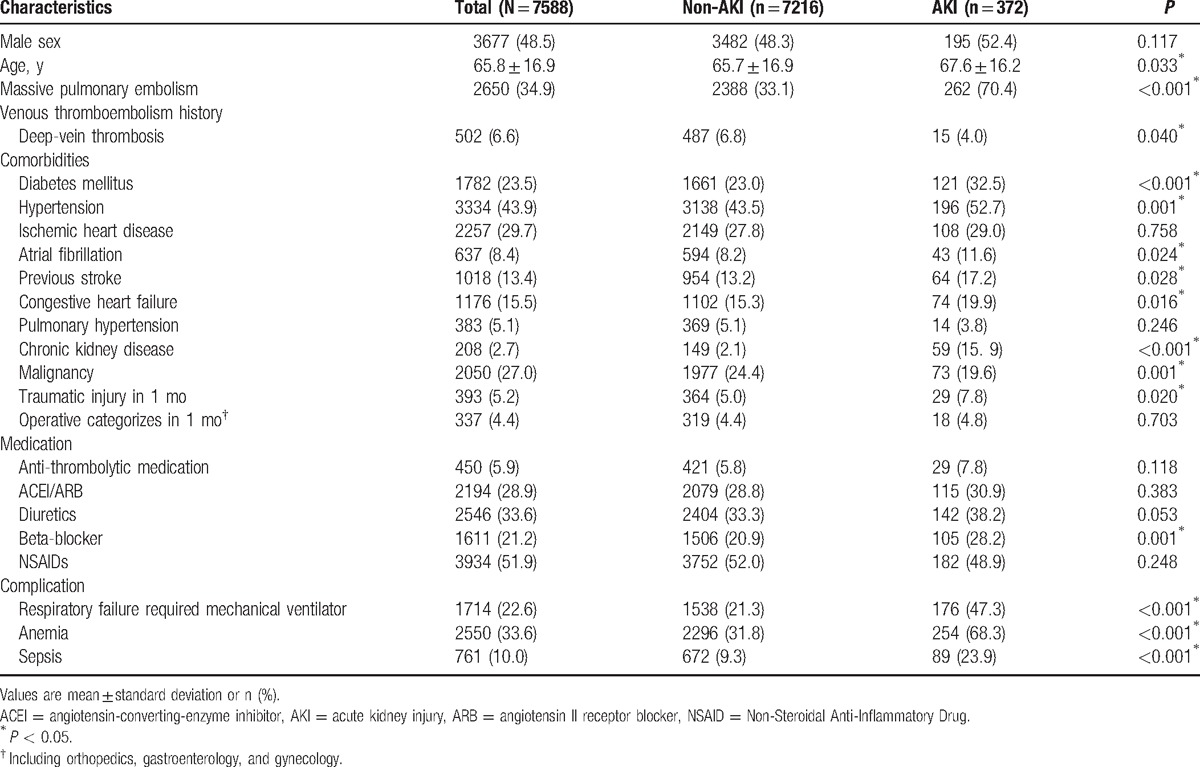
Clinical characteristics of patients who experienced pulmonary embolism and were administered anticoagulants for acute kidney injury.

## 3 Results

### 3.1 Patient characteristics

We enrolled a total of 7588 adults (3677 men and 3911 women), with a mean age of 65.8 years. AKI was diagnosed in 372 (4.9%) patients, of which 174 (46.8%) received hemodialysis and 201 (54.0%) died during hospitalization. A history of DVT (deep vein thrombosis) was recorded in 6.6% of patients during enrollment. The studied patient characteristics included age, sex, comorbidities, and medications, as listed in Table 1. Patients in the AKI group were significantly older and exhibited a markedly higher prevalence of the following comorbidities: HF, atrial fibrillation, previous MI, CKD, previous stroke, hypertension, diabetes mellitus (DM), and traumatic injury. However, patients in the AKI group had a lower prevalence of malignancy; these patients had a higher incidence of massive PE, concomitant anemia, or sepsis and required ventilator support during hospitalization.

### 3.2 Factors associated with risk of acute kidney injury after pulmonary embolism

Table 2 summarizes the risk factors for AKI according to multivariable logistic regression. The results revealed that histories of CKD, hypertension, DM, massive PE, anemia, and sepsis during hospitalization were associated with a higher risk of AKI. However, malignancy occurrence was less likely in the AKI group (odds ratio [OR], 0.66; 95% confidence interval [CI], 0.50–0.87).

**Table 2:**
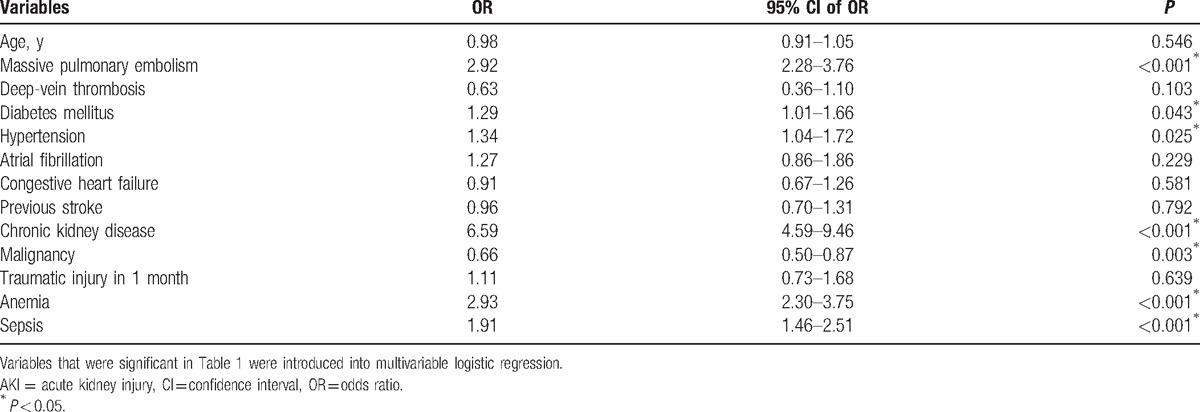
Factors associated with risks of postoperative AKI.

Short-Term Events in the Acute Kidney Injury and Non-Acute Kidney Injury Groups

A total of 1716 (22.6%) patients died before discharge, of which 1515 (21.0%) and 201 (54.0%) were in the non-AKI and AKI groups, respectively (Table 3). Patients in the AKI group exhibited significantly higher in-hospital mortality (OR, 2.62; 95% CI, 2.02–3.41), longer ICU stay (B, 1.63; 95% CI, 1.55–1.71), and longer hospital stays (B, 1.77; 95% CI, 1.73–1.82) than did those in the non-AKI group. However, no group differences were observed considering the risk of cardiovascular events, including HF, MI, and pulmonary hypertension, and major bleeding.

**Table 3:**
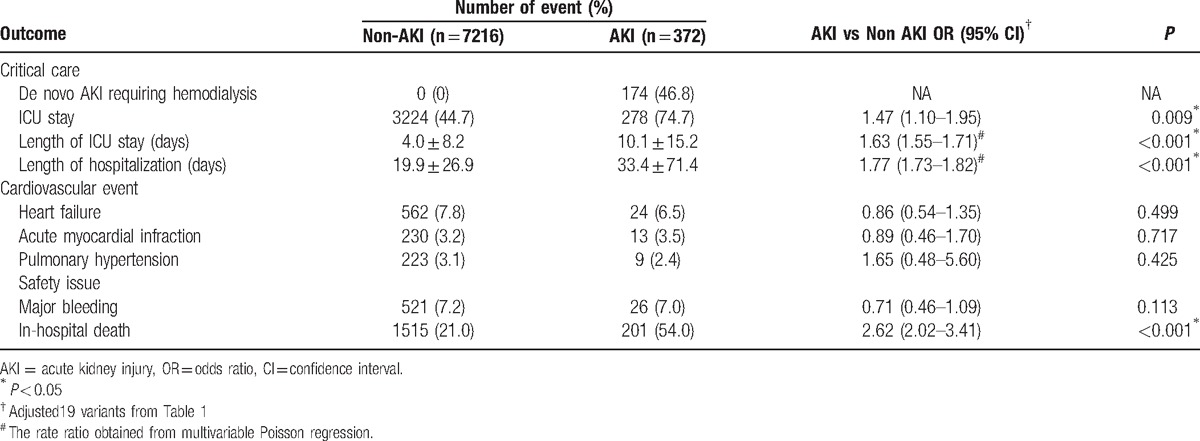
Short-term outcomes.

### 3.3 Outcomes at 1-year follow-Up

**Figure 2:**
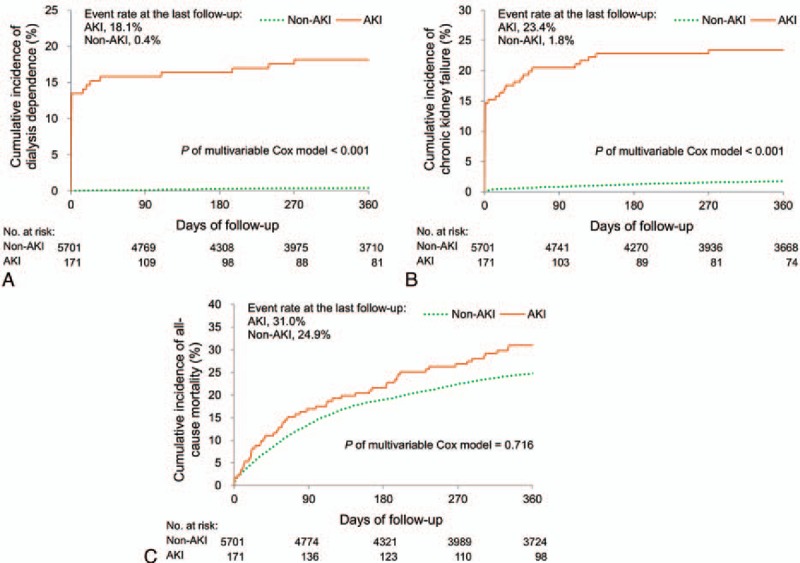
Cumulative incidence of 1-year outcomes in AKI and non-AKI groups: (A) Dialysis dependence, (B) CKD, and (C) all-cause mortality. AKI = acute kidney injury, CKD = chronic kidney disease.

We excluded 1716 patients who died during the index hospitalization and further compared the 1-year follow-up outcomes between the groups (Table 4). Overall, 1472 (25.1%) patients died within 1 year. One-year mortality rates in the non-AKI and AKI groups were 24.9% and 31.0% but not significantly different (hazard ratio [HR], 1.05; 95% CI, 0.79–1.40). Nevertheless, the risk of dialysis dependence was markedly higher in the AKI group than in the non-AKI group (18.1% vs 0.4%; HR, 7.62; 95% CI, 3.44–16.85). Similar results were observed for new-onset CKD. However, the risk of DVT, PE, pulmonary hypertension, HF, and major bleeding events did not vary between the groups. The Kaplan–Meier survival curves of dialysis dependence, new-onset CKD, and all-cause mortality is depicted in Fig. 2A–C, respectively.

**Table 4:**
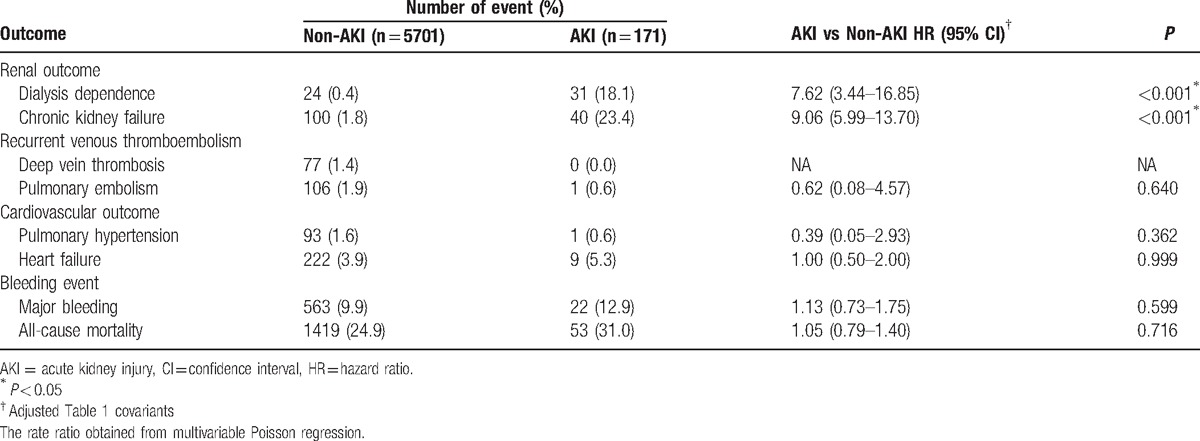
Outcomes at 1-year follow-up of patients who survived during the index admission.

## 4 Discussion

A U.S. database reported doubling in the incidence of PE from 62 cases per 100,000 in the 5-year period before 1998 to 112 cases per 100,000 in the 7 years after 1998.^[11]^ The incidence increased with age in women aged more than 70 years; the incidence rate was 2.75 per 1000 person-years.^[12]^ In Taiwan, PE remained a fatal disease, with an incidence rate of 3.18 cases per 100,000 in 2011.Kidney damage is not rare in PE. In our study, the AKI incidence was 4.9% and the OR of AKI-related mortality was 2.62 (95% CI, 2.02–3.41), which were similar to previous studies.

AKI is highly associated with mortality and treatment for patients complicated with AKI is challenging; therefore, risk factor analysis is particularly crucial for this condition. No previous study has analyzed the risk factors for AKI comorbid with PE. In this study, we found massive PE, sepsis, anemia, DM, hypertension, and CKD to be strong predictors of renal dysfunction. PE is a unique cause of HF and leads to cardiorenal syndrome, typically exhibiting complex syndromes with numerous pathways that affect renal function.^[13]^ Although the pathophysiology is not well understood, 4 possible explanations exist. First, in patients with massive PE, pulmonary hypertension and right HF will occur. An unstable hemodynamic status, hypoperfusion, and hypoxia can lead to renal dysfunction. Second, underlying diseases, such as DM, hypertension, and CKD, are risk factors for AKI that make the kidneys vulnerable during acute stress. Third, PE after traumatic injury might cause third-space fluid loss which might induce the prerenal azotemia. Fourth, concomitant conditions, such as sepsis, respiratory failure, and anemia, will contribute to renal dysfunction. Nevertheless, patients with PE comorbid with malignancy were shown to have a protective factor against AKI in our study, possibly because of their early diagnosis and treatment. Although PE results in AKI, Al-Dorzi reported that AKI itself is not a risk factor for PE.^[14]^

No prior research has mentioned the long-term outcomes of patients with PE complicated with AKI. Notably, we observed no remarkable differences in all-cause mortality, cardiovascular events, and major bleeding between patients with and without AKI at the 1-year follow-up. This result implies that intensive treatment and renal replacement therapy are warranted to protect the vital organs and might markedly change the outcomes. Nonetheless, in our study, renal outcomes were significantly poor in the AKI group, which is compatible with other causes of kidney injury.^[15,16]^ Patients became dialysis dependent and developed CKD. According to our review of the relevant literature, this study is the first to perform outcome analysis with risk factor evaluation. Intensive treatment was required to effectively treat patients with PE.

## 5 Limitations

This study has several limitations. First, the NHIRD did not include data on clinical factors, such as serum Cr. Thus, AKI staging and CKD severity were not provided. However, previous studies have reported the accuracy and admissibility of AKI and CKD diagnoses in the NHIRD, and the accuracy of CIC registration in the NHIRD has also been validated.

## 6 Conclusion

In the present study, we identified the predictors of AKI. Early recognition of these predictors might improve the accuracy of the clinical decision for early intervention. Accordingly, careful screening for medication, choice of therapy, and early intervention in patients showing increased biomarker levels were warranted for clinical care. The novelty of the present study is that it provides reliable evidence that patients have equal survivability after PE, although renal outcomes are poor in patients with AKI.
